# Osteogenic Effects of *Limosilactobacillus fermentum* GBE18 Cell-Free Supernatant (CFS) in MC3T3-E1 Cells via the Wnt/β-Catenin and PI3K/Akt Signaling Pathways

**DOI:** 10.3390/foods15081349

**Published:** 2026-04-13

**Authors:** Xingyuan Peng, Xuan Zheng, Xiyu Li, Xiaona Pang, Junhua Jin, Hui Liu, Hongxing Zhang, Yuanhong Xie

**Affiliations:** Beijing Laboratory of Food Quality and Safety, Beijing Key Laboratory of Agricultural Product Detection and Control of Spoilage Organisms and Pesticide Residue, College of Food Science and Engineering, Beijing University of Agriculture, Beijing 102206, China

**Keywords:** osteoporosis, *Limosilactobacillus fermentum*, cell-free supernatant, osteogenic differentiation, Wnt/β-Catenin pathway, PI3K/Akt pathway

## Abstract

Osteoporosis is a major global health challenge, particularly among aging populations, underscoring the need for safe and effective nutritional interventions. Probiotics and their metabolites have emerged as promising candidates for modulating bone health via the gut-bone axis. In this study, we investigated the effects of a cell-free culture supernatant (CFS) from the food-grade bacterium *Limosilactobacillus fermentum* GBE18 on the proliferation, differentiation, and mineralization of MC3T3-E1 pre-osteoblasts. GBE18 CFS exhibited no cytotoxicity at concentrations ranging from 1% to 4% (*v*/*v*). Notably, 2% (*v*/*v*) CFS significantly enhanced alkaline phosphatase (ALP) activity and extracellular matrix mineralization (*p* < 0.05). Transcriptomic profiling revealed that differentially expressed genes were enriched in osteoblast-related processes and two key signaling pathways: Wnt/β-catenin and PI3K/Akt. Subsequent qRT-PCR and Western blot analyses confirmed the upregulation of critical regulators (*Rspo2*, *Pdpk1*, *Malat1*) and demonstrated coordinated activation of Akt phosphorylation, β-catenin stabilization, and Runx2 protein expression. Our findings indicate that GBE18 CFS promotes osteogenic differentiation through coordinated modulation of the PI3K/Akt and Wnt/β-catenin pathways. Consequently, this study provides mechanistic evidence supporting the potential application of *L. fermentum* GBE18-derived metabolites as functional food ingredients or dietary interventions for bone health and osteoporosis management.

## 1. Introduction

Osteoporosis is a widespread chronic metabolic bone disorder that predominantly affects the elderly population worldwide [1]. This condition is characterized by reduced bone mass and density, microarchitectural deterioration, increased skeletal fragility, and a heightened risk of fractures [2]. Current clinical interventions, including anti-resorptive agents like bisphosphonates and anabolic treatments such as parathyroid hormone analogs, aim to mitigate bone loss and reduce fracture risk [3]. However, the prolonged administration of these pharmacological agents is frequently associated with significant adverse effects. Specifically, calcium supplementation may lead to hypercalcemia, bisphosphonates can cause gastrointestinal disturbances, and hormone replacement therapy has been linked to an increased risk of breast cancer [4,5,6]. Therefore, there is an urgent need for the development of novel, safer therapeutic or preventive strategies. While traditional anti-resorptive agents focus on mitigating bone loss, anabolic strategies, which enhance bone mass by promoting osteoblast proliferation and differentiation, represent a more proactive and promising approach.

Probiotics, defined as live microorganisms that confer health benefits on the host when administered in adequate amounts [7], have gained significant attention following the emergence of the “gut-bone axis” concept, which identifies the gut as a novel therapeutic target for osteoporosis. Mechanistically, dysregulation of the gut-bone axis may contribute to osteoporosis by altering nutrient absorption, intestinal barrier function, immune homeostasis, and systemic inflammatory signaling, thereby disrupting the balance of bone remodeling. Comparative studies reveal substantial differences in microbial diversity between patients with osteoporosis and healthy individuals, demonstrating that the severity of bone loss correlates with microbiota composition [8,9]. Regarding therapeutic applications, clinical research involving elderly women indicates that probiotic supplementation can effectively mitigate bone loss [10]. A large body of preclinical research in animal models has established the bone-protective effects of probiotics. For instance, the study by Montazeri-Najafabady et al. demonstrated that 16-week intervention with *Bifidobacterium longum* in ovariectomized rats significantly increased bone mineral density, improved trabecular microarchitecture, and suppressed osteoclast activity [11]. Furthermore, Seijo et al. reported that yogurt enriched with *Lacticaseibacillus casei*, *Limosilactobacillus reuteri*, and *Ligilactobacillus gasseri* effectively enhanced intestinal calcium absorption and increased bone mineral content in vivo [12]. In addition, the research conducted by Guo et al. revealed that *Lacticaseibacillus rhamnosus* GG ameliorates estrogen deficiency-induced osteoporosis by modulating gut microbiota, intestinal barrier function, and the Th17/Treg balance [13]. At the cellular level, extracts from *Lacticaseibacillus paracasei* L30 and strains such as *Lactobacillus acidophilus* promote the differentiation and maturation of osteoblasts [14,15]. Consequently, incorporating probiotics into the daily diet represents a promising strategy for the prevention and mitigation of osteoporosis.

Previous studies have shown that certain *L. fermentum* strains possess probiotic activities, and have been reported to exert bone-protective effects in osteoporotic animal models. However, the direct osteogenic effects of probiotic-derived soluble factors and their underlying mechanisms remain poorly understood. Based on a preliminary screening of 38 food-related probiotic strains preserved in our laboratory, using osteoblast mineralization as the primary evaluation index, *L. fermentum* GBE18 was identified as a candidate strain with prominent pro-osteogenic activity and was therefore selected for further study. *Limosilactobacillus fermentum* is a lactic acid bacterium widely used in food fermentation and probiotic research. In the present study, we investigated the pro-osteogenic effects of the cell-free supernatant (CFS) derived from *Limosilactobacillus fermentum* GBE18 on MC3T3-E1 cells and further elucidated the underlying molecular mechanisms. These findings provide insights into the mechanistic basis of the pro-osteogenic properties of GBE18 CFS, and offer experimental support for understanding the role of probiotic-derived metabolites in bone health regulation.

## 2. Materials and Methods

### 2.1. Materials and Reagents

Osteoblast differentiation medium (ODM) and α-Minimum Essential Medium were obtained from Procell Biotechnology Co., Ltd. (Wuhan, Hubei, China). Phosphate-buffered saline (PBS), trypsin-EDTA, CCK-8 assay kit, and Alizarin Red S were purchased from Solarbio Science & Technology Co., Ltd. (Beijing, China). The BCA protein concentration assay kit was sourced from Beyotime Biotechnology Co., Ltd. (Shanghai, China), and the alkaline phosphatase (ALP) assay kit was obtained from Jiancheng Bio-engineering Institute (Nanjing, Jiangsu, China). Triton X-100 cell lysis buffer was supplied by Yuanye Bio-Technology Co., Ltd. (Shanghai, China). 24-well plates were acquired from Lanjieke Technology Co., Ltd. (Beijing, China). TRIzol reagent and monoclonal antibodies were sourced from Aladdin Biochemical Technology Co., Ltd. (Shanghai, China) and Servicebio Technology Co., Ltd. (Wuhan, Hubei, China), respectively.

### 2.2. Bacterial Strain and Preparation of Cell-Free Supernatant (CFS)

*Limosilactobacillus fermentum* GBE18 was isolated from Guangxi rice noodles and identified using full-length 16S rRNA gene sequencing. *L. fermentum* GBE18 was activated through two successive passages and then inoculated at a 2% (*v*/*v*) concentration into de Man, Rogosa, and Sharpe (MRS) medium, followed by incubation for 20 h at 37 °C. The culture was then centrifuged at 8000 rpm for 10 min at 4 °C. The pH of the resulting supernatant was adjusted to 7.3 with 1 mol/L NaOH, sterilized using a 0.22 μm sterile filter membrane to obtain the CFS, and subsequently stored at −80 °C.

### 2.3. Cell Culture and Osteogenic Induction

As a well-established osteoblast precursor cell line, MC3T3-E1 cells exhibit biological functions comparable to those of primary osteoblasts [16]. Their robust proliferative and osteogenic potential makes them an ideal model for investigating the intricate processes of osteogenic differentiation [17].

The MC3T3-E1 cell line was purchased from Wuhan Procell Biotechnology Co., Ltd. (Wuhan, Hubei, China). These cells were maintained in α-Minimum Essential Medium (α-MEM) supplemented with 10% fetal bovine serum (FBS), 100 units/mL penicillin, and 100 μg/mL streptomycin at 37 °C in a 5% CO_2_ incubator. When the cells reached approximately 80% confluence, they were either passaged or cryopreserved for subsequent use.

For osteogenic differentiation experiments, upon reaching 80% confluence, the culture medium was replaced with osteogenic differentiation medium (ODM). The ODM was formulated with the same basal α-MEM used for routine cell culture, supplemented with 10% FBS, 1% penicillin-streptomycin, 10 mM β-glycerophosphate, 50 μg/mL ascorbic acid, and 100 nM dexamethasone, all purchased from Wuhan Procell Biotechnology Co., Ltd. (Wuhan, Hubei, China). The ODM was refreshed every 3 days throughout the induction period.

### 2.4. Cell Proliferation Assay

The impact of GBE18 CFS on the proliferation of MC3T3-E1 cells was assessed using the CCK-8 assay kit (Solarbio, Beijing, China). MC3T3-E1 cells were initially seeded at a density of 2 × 10^4^ cells/mL in 96-well plates and cultured in 100 μL of α-MEM for 24 h. Subsequently, the medium was replaced with ODM containing 1%, 2%, or 4% (*v*/*v*) GBE18 CFS, while cells cultured in α-MEM alone were employed as the control (CK) group. Following incubation periods of 24, 48, and 72 h, 10 μL of CCK-8 solution was added to each well, and the plates were incubated for an additional 2 h at 37 °C in the absence of light. Absorbance was then measured at 450 nm using a SpectraMax^®^ Mini multi-mode microplate reader(Molecular Devices, San Jose, CA, USA). The proliferation ratio (W) was determined using the following formula:

W(%) = (A_sample_ − A_blank_)/A_blank_ × 100%.

### 2.5. Quantitative Detection of ALP Activity

MC3T3-E1 cells were initially seeded into 24-well plates (Lanjieke, Beijing, China) at a density of 8 × 10^4^ cells per well, with each well containing 500 µL of α-MEM. Cells typically reached approximately 80% confluence after 4 days of culture. At that point, the CK group continued to be cultured in α-MEM, whereas the other wells were exposed to an osteogenic differentiation medium (ODM) supplemented with varying concentrations of GBE18 CFS at 0% (ODM), 1%, 2%, or 4% (*v*/*v*). The culture medium was replaced every 3 days during the 14-day incubation period. Following the incubation period, the medium was carefully aspirated, and the wells were thoroughly washed with PBS (Solarbio, Beijing, China) to eliminate any residual serum. Subsequently, cell lysis was performed by adding 100 μL of Triton X-100 lysis buffer (Yuanye, Shanghai, China) to each well, followed by incubation on ice for 1 h. The total protein concentration was measured using a BCA protein assay kit (Beyotime, Shanghai, China), and alkaline phosphatase (ALP) activity was assessed using an ALP assay kit (Jiancheng, Nanjing, China), according to the manufacturer’s instructions. For comparative analysis across different groups, ALP activity was normalized to the total protein content.

### 2.6. Alizarin Red S Staining Mineralization Experiment

Alizarin Red S staining was utilized to evaluate mineralization levels. MC3T3-E1 cells, at a density of 3 × 10^5^ cells per well, were seeded into 6-well plates. The cells were then treated with α-MEM (CK group), ODM alone (ODM group), or ODM supplemented with 1%, 2%, or 4% (*v*/*v*) GBE18 CFS (GBE18 groups). The medium was replaced every 3 days throughout the 21-day culture period. After 21 days of culture, the cells were washed with phosphate-buffered saline (PBS), fixed with a 4% paraformaldehyde solution for 30 min, and subsequently incubated with a 1% Alizarin Red S staining solution (pH 4.2, Solarbio, Beijing, China) at room temperature for 30 min. Following extensive washing with PBS, images were captured using an optical microscope. The area of mineralized nodules was quantified using ImageJ software (version 1.54p).

### 2.7. Transcriptomic Sequencing

MC3T3-E1 cells were seeded into 24-well plates at a density of 8 × 10^4^ cells per well and divided into two experimental groups: the ODM group, cultured in ODM, and the GBE18 group, cultured in ODM supplemented with 2% GBE18 CFS. Both groups were maintained under these conditions for a duration of 14 days. Each group included three independent biological replicates (*n* = 3) for RNA sequencing. To minimize variation prior to RNA extraction, both groups were seeded at the same initial density and harvested at the same time point. Cell pellets containing approximately 1 × 10^6^ cells were collected from each sample for RNA extraction. Post-cultivation, cells underwent washing with phosphate-buffered saline (PBS) and trypsinization (Soarbio, Beijing, China), followed by collection and centrifugation at 1000 rpm for 5 min at 4 °C. The resultant cell pellets, containing approximately 1 × 10^6^ cells, were resuspended in 1 mL of TRIzol^®^ reagent (Aladdin, Shanghai, China)and homogenized through repeated pipetting to facilitate total RNA extraction. For the purpose of eukaryotic mRNA sequencing, library construction was performed using the Illumina^®^ Stranded mRNA Prep, Ligation Kit (Illumina, San Diego, CA, USA). RNA concentration and purity were measured using a Nanodrop 2000 spectrophotometer (Thermo Fisher Scientific, Waltham, MA, USA), while RNA integrity was assessed via agarose gel electrophoresis, and RNA Integrity Number (RIN) values were determined using an Agilent 5300 system (Agilent Technologies, Santa Clara, CA, USA). Subsequently, double-stranded complementary DNA (cDNA) was synthesized using fragmented mRNA as a template and random primers. The process involved end repair, A-tailing, and sequencing adapter ligation, after which the ligation products were purified, size-selected, and PCR-amplified to generate the sequencing library. The library was quantified utilizing a Qubit 4.0 Fluorometer (Thermo Fisher Scientific, Waltham, MA, USA) prior to undergoing bridge PCR amplification via the cBot system (Illumina, CA, USA). Subsequently, high-throughput sequencing was conducted on the NovaSeq X Plus platform (Illumina, CA, USA) using the NovaSeq Reagent Kit, in a paired-end 150 bp (PE150) configuration.

### 2.8. Real-Time Fluorescent Quantitative Reverse Transcription PCR

Cell culture procedures were conducted as outlined in Section 2.6. Total RNA was isolated utilizing the TiGen Total RNA Prep Pure Kit (DP430, TIANGEN, Beijing, China). The PCR primers were synthesized by Beijing Qingke Biotechnology Co., Ltd. (Beijing, China), with the following sequences: Rspo2-F (GCCCGTCATTTCCTCGACTT), Rspo2-R (GGGATTTGATACATAACTAGCTCGC); Pdpk1-F (ATGGGTCCAGTGGATAAGCG), Pdpk1-R (CCGGTAATTACATCGTGTGGA); Malat1-F (TGTGACGCGACTGGAGTATG), Malat1-R (CAAAGGGACTCGGCTCCAAT); Gapdh-F (GCAAAGTGGAGATTGTTGCCAT), Gapdh-R (CCTTGACTGTGCCGTTGAATTT). Complementary DNA (cDNA) synthesis was carried out using the FastKING One-Step Reverse Transcription-qPCR Kit (FP313-01, TIANGEN, Beijing, China), followed by a two-step real-time quantitative PCR analysis on an Applied Biosystems 7500 system (Thermo Fisher Scientific, Waltham, MA, USA). The expression of target genes was normalized to Gapdh, and relative expression levels were determined using the 2^−ΔΔCt^ method.

### 2.9. Western Blot

MC3T3-E1 cells were seeded into 6-well plates at a density of 3 × 10^5^ cells/mL and cultured in α-MEM for 48 h. Subsequently, the culture medium was replaced with ODM containing 2% (*v*/*v*) GBE18 CFS. After 14 days of culture, the cells were lysed using RIPA buffer supplemented with phosphatase and protease inhibitors. Protein concentrations were quantified utilizing a bicinchoninic acid (BCA) protein assay kit. Equivalent amounts of protein lysates were subjected to separation on a 10% SDS-PAGE gel and subsequently transferred onto a polyvinylidene difluoride (PVDF) membrane. The membranes were blocked with 3% bovine serum albumin (BSA) for 2 h, washed with Tris-buffered saline containing 0.1% Tween 20 (TBST), and incubated overnight at 4 °C with primary antibodies. The primary antibodies used included anti-phospho-Akt, anti-total Akt, anti-active β-catenin, and anti-Runx2 monoclonal antibodies. Finally, the membranes were incubated with enzyme-labeled secondary antibodies, and protein bands were detected using enhanced chemiluminescence and quantified using Quantity One software (Version 4.6.6).

### 2.10. Statistical Analysis

Data are presented as mean ± standard deviation (SD). Statistical analyses were performed using GraphPad Prism software (version 9.5.1). Data distribution was assessed before applying parametric statistical methods. Differences between two independent groups were evaluated using an unpaired Student’s *t*-test, while multiple group comparisons were analyzed via one-way analysis of variance (ANOVA). Following ANOVA, Dunnett’s post-hoc test was applied for comparisons between each treatment group and the control group, provided that homogeneity of variance was met. Statistical significance was defined as a two-tailed *p* < 0.05.

## 3. Results

### 3.1. Effects of GBE18 on MC3T3-E1 Cell Proliferation

**Figure 1:**
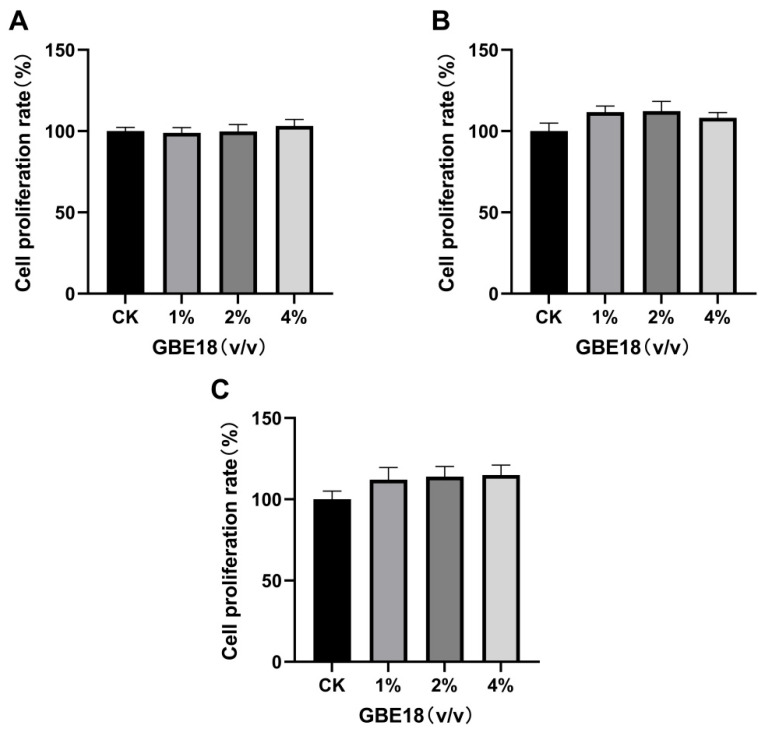
Effects of various GBE18 concentrations on the proliferation of MC3T3-E1 cells. Proliferation was assessed after (**A**) 24 h, (**B**) 48 h, and (**C**) 72 h of GBE18 treatment. Data are presented as mean ± SD (*n* = 6).

The results of the CCK-8 assay demonstrated that after 24 h (Figure 1A) and 48 h (Figure 1B) of treatment with 1%, 2%, and 4% (*v*/*v*) GBE18 CFS, there were no statistically significant differences in relative cell viability compared to the control (CK) group (*p* > 0.05). However, after 72 h of treatment, relative cell viability, as indicated by the CCK-8 signal, increased by approximately 14% relative to the control group (Figure 1C). These findings indicate that GBE18 CFS is non-cytotoxic to MC3T3-E1 cells across the tested concentration range.

### 3.2. The Effect of GBE18 on the Differentiation of MC3T3-E1 Cells

**Figure 2:**
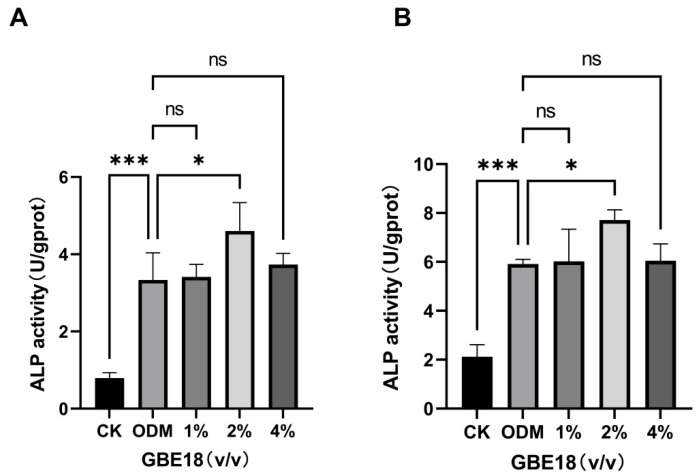
Effects of varying GBE18 concentrations on ALP activity in MC3T3-E1 cells. (**A**) ALP activity at 7 days and (**B**) 14 days of treatment. Group definitions: CK group: cells cultured in basal α-MEM without osteogenic induction; ODM group: cells cultured in osteogenic differentiation medium (ODM) without GBE18 CFS treatment; 1%, 2%, 4% groups: cells cultured in ODM supplemented with 1%, 2%, 4% (*v*/*v*) GBE18 CFS, respectively. ALP activity was normalized to the total protein content of each sample, and expressed as units per gram of protein (U/g protein). Data are presented as mean ± SD (*n* = 3). Statistical analysis was performed using one-way analysis of variance (ANOVA) followed by Dunnett’s post hoc test. * *p* < 0.05, *** *p* < 0.001 vs. ODM group; ns, non-significant.

Seven days post-osteogenic induction, alkaline phosphatase (ALP) activity in the CK group was 0.7 U/g protein, whereas the ODM group reached 3.3 U/g protein. This significant increase in the ODM group relative to the CK group (*p* < 0.001) confirmed the successful induction of osteoblast differentiation. Treatment with 2% (*v*/*v*) GBE18 CFS led to a 1.3-fold increase in ALP activity in MC3T3-E1 cells compared to the ODM group, significantly exceeding the positive control (*p* < 0.05) (Figure 2A). No significant differences were observed in the 1% and 4% groups compared to the ODM group, indicating that 2% (*v*/*v*) GBE18 CFS was the most effective concentration for promoting osteogenic differentiation. By day 14 of induction, ALP activity in the 2% (*v*/*v*) group had substantially increased to 7.7 U/g protein from the 4.6 U/g protein recorded at day 7. The intergroup trends observed at day 7 persisted. ALP activity remained significantly lower in the CK group than in both the ODM and CFS groups (*p* < 0.001), while the CFS group exhibited significantly higher activity than the ODM group (*p* < 0.05) (Figure 2B).

### 3.3. Effect of GBE18 on Mineralization in MC3T3-E1 Cells

**Figure 3:**
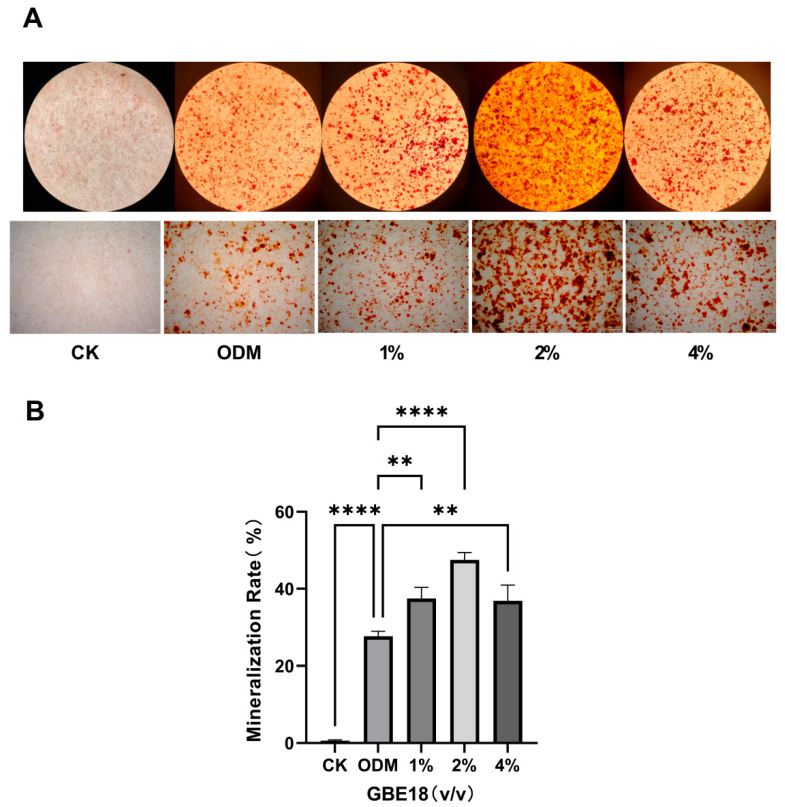
Effects of GBE18 on mineralization in MC3T3-E1 cells. (**A**) Representative images of Alizarin Red S-stained MC3T3-E1 cells cultured under the indicated conditions, acquired under light microscopy (magnification, ×40). More intense Alizarin Red S staining and increased mineralized nodule formation indicate enhanced matrix mineralization. (**B**) Quantitative analysis of Alizarin Red S staining in MC3T3-E1 cells from the indicated groups. Data are presented as mean ± SD (*n* = 3). Statistical significance was determined using one-way ANOVA followed by Dunnett’s post hoc test. ** *p* < 0.01, **** *p* < 0.0001 vs. ODM group.

Extracellular matrix calcification was evaluated via Alizarin Red S staining to assess late-stage osteogenic differentiation. After 21 days of culture in osteogenic differentiation medium (ODM), all experimental groups were subjected to Alizarin Red S staining (Figure 3A). While the CK group exhibited minimal calcium nodule formation, the ODM group showed pronounced mineralized nodules, confirming the efficacy of ODM in inducing MC3T3-E1 cell mineralization. Groups treated with varying concentrations of GBE18 CFS demonstrated significant osteogenic mineralization; notably, the 2% (*v*/*v*) group exhibited significantly enhanced mineralization compared to the ODM group, characterized by abundant calcium nodules. Quantitative analysis using ImageJ software (Figure 3B) revealed that the mineralization rate in the ODM group was 27% higher than that in the CK group (*p* < 0.0001). Furthermore, the 2% GBE18 group showed a 20% increase in mineralization relative to the ODM group, with significantly enhanced calcium deposition (*p* < 0.0001). These findings were consistent with the ALP activity assays, leading to the selection of the 2% (*v*/*v*) concentration for subsequent experiments.

### 3.4. Transcriptomic Data Analysis of the Mechanism of Action of GBE18 on MC3T3-E1 Cells

**Figure 4:**
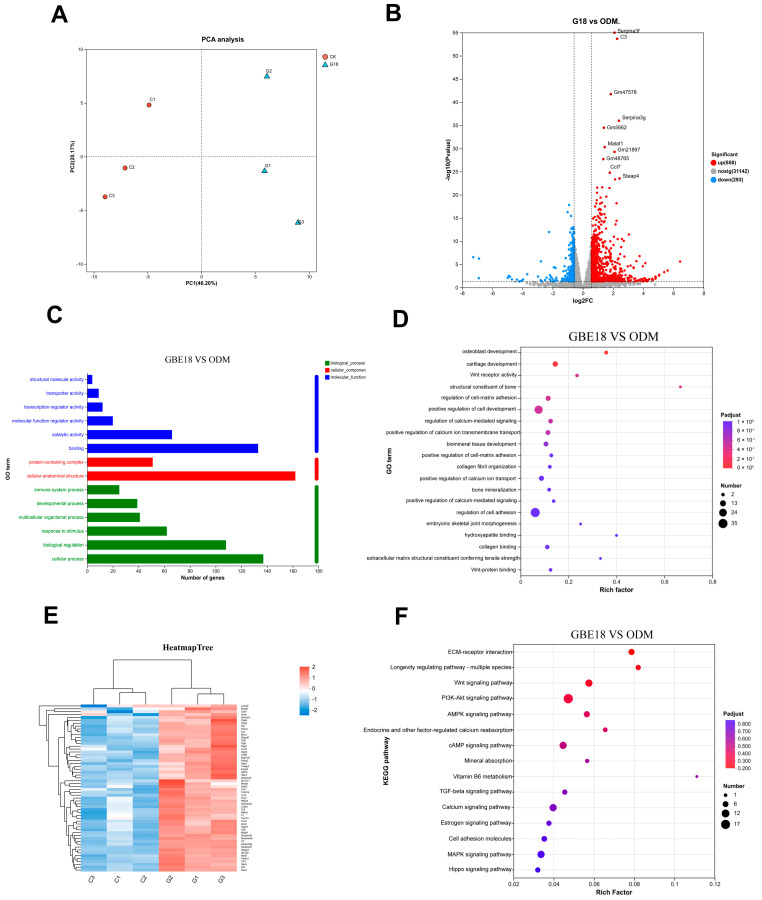
Comparative analysis of differentially expressed genes (DEGs) in MC3T3-E1 cells treated with GBE18 versus osteogenic differentiation medium (ODM) at day 14. (**A**) Principal component analysis (PCA) plot illustrating the global transcriptional variation between groups. (**B**) Volcano plot of DEGs identified in the GBE18 group relative to the ODM group. Significantly upregulated genes are indicated in red, downregulated genes in blue, and non-differentially expressed genes in grey. (**C**) Gene Ontology (GO) functional classification of DEGs between the GBE18 and ODM groups. (**D**) Heatmap depicting the expression patterns of selected osteogenesis-related DEGs derived from the GO classification (red: upregulated; blue: downregulated). (**E**) GO enrichment analysis of significantly altered osteogenesis-related DEGs. (**F**) KEGG pathway analysis highlighting significantly upregulated osteogenesis-related DEGs. RNA-seq analysis was performed using three independent biological replicates per group (*n* = 3).

To elucidate the molecular mechanisms underlying the pro-osteogenic effects of GBE18 on MC3T3-E1 cells, RNA sequencing (RNA-seq) was performed on osteoblasts treated with 2% (*v*/*v*) CFS from the GBE18 group and the ODM group. Principal component analysis (PCA) revealed a distinct separation between the GBE18 and ODM samples along the PC1 axis (Figure 4A), indicating significant transcriptional remodeling induced by the treatment. Volcano plot analysis identified 1143 differentially expressed genes (DEGs) in the GBE18 group compared to the ODM group, comprising 850 significantly upregulated and 293 significantly downregulated genes. Notably, the long non-coding RNA (lncRNA) *Malat1* (metastasis-associated lung adenocarcinoma transcript 1) was markedly upregulated in the GBE18-treated group (Figure 4B).

Differentially expressed genes (DEGs) between the GBE18 and ODM groups were identified using a screening threshold of |log_2_FC| ≥ 1 and *p* < 0.05. To characterize the functional distribution of these genes, Gene Ontology (GO) Level 2 annotation analysis was performed, encompassing three primary categories: biological processes (BP), cellular components (CC), and molecular functions (MF) (Figure 4C). Within the biological process category, DEGs were predominantly enriched in cellular processes and biological regulation, alongside involvement in osteogenesis-related developmental and multicellular processes. This suggests that these DEGs may play a pivotal role in regulating osteoblast differentiation and function. Regarding cellular components, DEGs were primarily localized to cellular anatomical structures, followed by protein-containing complexes. This distribution indicates that the products of these DEGs are mainly situated within cellular frameworks or protein assemblies, facilitating functional execution at the cellular level. In terms of molecular functions, binding and catalytic activities emerged as the predominant functional categories. These attributes align with the enzymatic requirements for signal transduction and matrix synthesis during osteogenesis.

To elucidate the core mechanisms underlying GBE18-mediated osteogenesis, we integrated these findings with previous GO functional annotation results. We specifically selected DEGs associated with secondary functional terms pertinent to osteogenesis, such as binding activity, developmental processes, biological regulation, and structural/molecular activity, for GO enrichment analysis. The results demonstrate that these DEGs are significantly enriched in biological processes and molecular functions closely linked to osteogenesis (Figure 4D). Notably, the core enriched biological process terms include osteoblast development, cartilage development, bone mineralization, collagen fiber organization, and regulation of cell-matrix adhesion. Core enriched molecular function terms comprised Wnt receptor activity, bone structural component, hydroxyapatite binding, and collagen binding.

We conducted further screening of 58 core upregulated genes from the pool of differentially expressed genes and subjected them to hierarchical clustering analysis (Figure 4E). These core upregulated genes span multiple functional dimensions that are intricately linked to osteogenesis. Notable upregulated genes include those involved in extracellular matrix (ECM) remodeling, such as *Mmp8*, *Mmp9*, *Dcn*, and *Has3*; genes that regulate osteoblast development and differentiation, including *Hipk2*, *Rspo2*, and *Cd69*; genes participating in signal transduction pathways, such as *Akap5*, *Rasgrp2*, and *Tlr2*; and genes associated with bone mineralization and cell survival, including *Saa3*, *Lcn2*, and *Enpp2*. Additionally, the upregulated genes include those with catalytic activity and other functions, exemplified by *Ggt5* and *Cxcl1*. The analysis of differentially expressed genes revealed two distinct core expression clusters. One cluster demonstrated significantly elevated expression levels in the GBE18 group, represented by the red region in the heatmap, while the other cluster exhibited high expression in the ODM group, indicated by the blue region. This suggests that treatment with GBE18 CFS specifically alters the gene expression profile of osteoblasts. Additionally, replicate samples from both the GBE18 and ODM groups formed distinct sub-clusters, displaying highly consistent gene expression patterns within each group. This consistency underscores the biological stability of the experimental replicates.

KEGG pathway analysis further confirmed the involvement of signaling pathways associated with osteogenesis. As illustrated in Figure 4F, pathways significantly enriched in relation to osteogenesis included ECM-receptor interaction, Wnt signaling, PI3K/Akt signaling, AMPK signaling, TGF-β signaling, and calcium signaling. Additionally, pathways involved in mineral absorption and endocrine-regulated calcium reabsorption were identified. These pathways are integral to regulating the proliferation and osteogenic differentiation of MC3T3-E1 cells, with the Wnt and PI3K/Akt signaling pathways being particularly well-established in this context. These findings suggest that GBE18 CFS may enhance osteogenesis by synergistically modulating multiple critical signaling pathways.

Among the genes listed in Table 1, *Rspo2* and *Fzd5* were identified as the most significantly enriched molecules. *Rspo2* demonstrated an exceptionally low enrichment *p*-value of 2.746 × 10^−11^, with a corresponding false discovery rate (FDR) of 3.95 × 10^−9^, which is substantially below the threshold of 0.05. This gene also shows significant upregulation in the treatment group, as evidenced by a Log_2_ fold change (Log_2_FC) of 1.0009 and a fold change of 2.0013. Similarly, *Fzd5* exhibited a high level of significance with an enrichment *p*-value of 7.990 × 10^−10^ and an FDR of 6.855 × 10^−8^. Functionally, *Rspo2* serves as a specific activator of the Wnt signaling pathway, whereas *Fzd5* functions as a transmembrane receptor within the same pathway. Another molecule of notable prominence is *Pdpk1*, which acts as a key downstream kinase in the PI3K/Akt signaling pathway. *Pdpk1* has an enrichment *p*-value of 4.447 × 10^−11^ and an FDR of 5.823 × 10^−9^, both well below the 0.05 threshold, and it is upregulated in the treatment group, with a Log_2_FC of 0.6922 and a fold change of 1.6157. The significant upregulation of these genes suggests effective activation of both the Wnt and PI3K/Akt pathways.

### 3.5. Effects of GBE18 on the Expression of Osteogenesis-Related Marker Genes and Proteins

**Figure 5:**
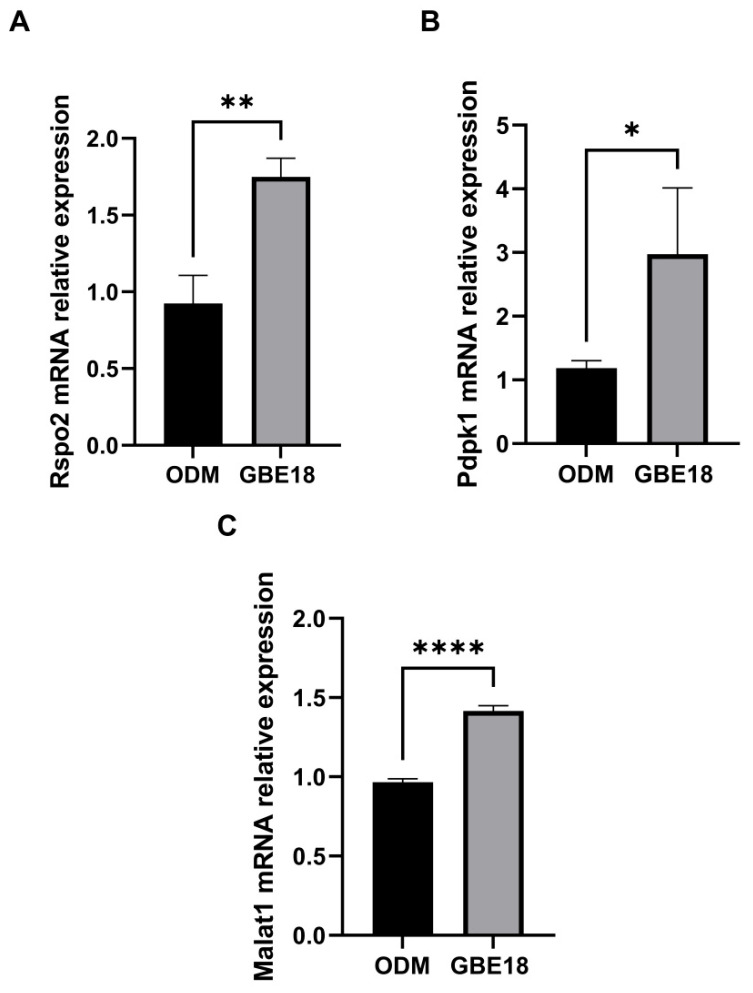
Effects of GBE18 on the expression of osteogenic differentiation-related genes in MC3T3-E1 cells after 14 days of treatment. Data are presented as mean ± SD (*n* = 3). (**A**–**C**) Relative mRNA expression levels of the osteogenic marker genes *Rspo2* (**A**), *Pdpk1* (**B**), and *Malat1* (**C**) were measured by RT-qPCR in GBE18-treated cells compared with the ODM group. Statistical analysis was performed using paired *t*-tests: * *p* < 0.05, ** *p* < 0.01, **** *p* < 0.0001.

Based on transcriptomic analyses, we validated key genes using quantitative reverse transcription PCR (qRT-PCR). The results indicated that, relative to the ODM group, the expression of *Rspo2* mRNA in the GBE18 group treated for 14 days was significantly upregulated to 1.89-fold (*p* < 0.01) (Figure 5A). Similarly, *Pdpk1* expression was increased to 2.5-fold (*p* < 0.05), with both genes reaching their highest expression levels in the GBE18 CFS group, showing statistically significant differences (Figure 5B). Furthermore, *Malat1* expression exhibited a 1.46-fold increase compared to the ODM group (Figure 5C), which was highly significant (*p* < 0.0001). These findings are consistent with the transcriptomic sequencing data, providing transcriptional evidence for the activating effects of GBE18 CFS on genes associated with osteogenic differentiation and the Wnt and PI3K signaling pathways.

**Figure 6:**
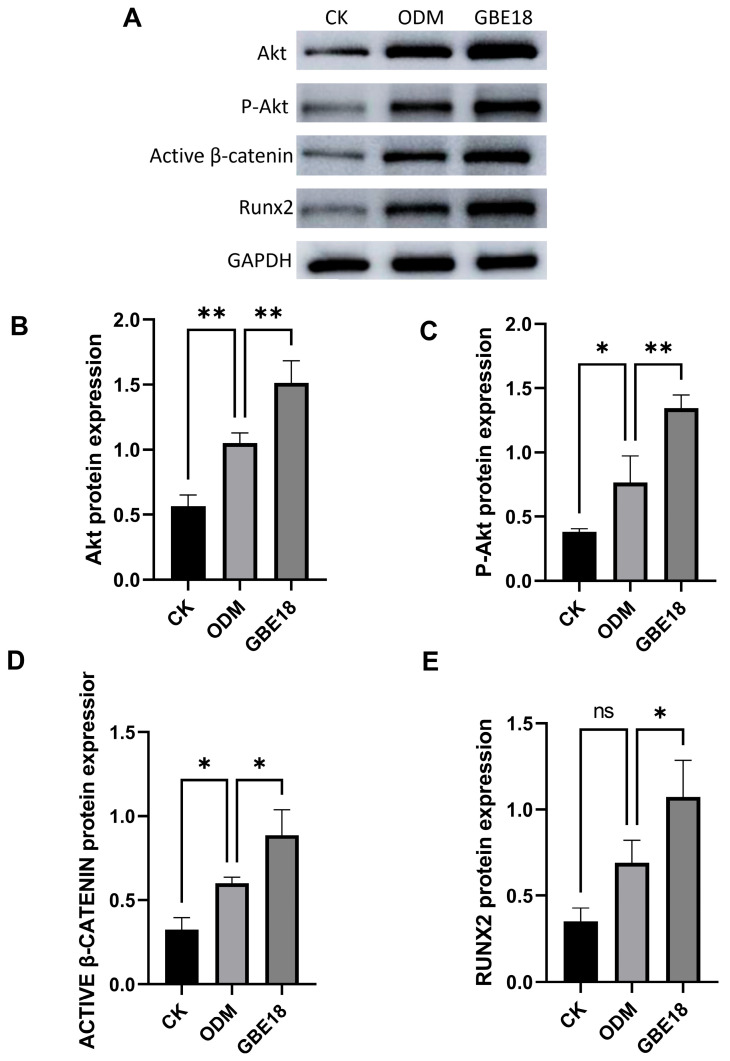
GBE18 modulates the expression of osteogenic differentiation-related proteins in MC3T3-E1 cells after 14 days of treatment. (**A**) Representative Western blot images illustrating the protein levels of Akt, p-Akt, active β-catenin, and Runx2. (**B**–**E**) Quantitative analysis of (**B**) Akt, (**C**) p-Akt, (**D**) active β-catenin, and (**E**) Runx2 protein expression. Data are presented as mean ± SD (*n* = 3). Statistical significance was determined using one-way ANOVA followed by Dunnett’s post hoc test relative to the ODM group: * *p* < 0.05, ** *p* < 0.001; ns, not significant.

Protein levels of phosphorylated Akt (p-Akt), total Akt, active β-catenin, and Runx2 were assessed using Western blot analysis, as illustrated in Figure 6A. Osteogenic differentiation medium significantly increased the levels of these proteins, whereas treatment with the GBE18 group resulted in maximal protein expression. Compared to the CK group, the ODM group demonstrated a significant upregulation of Akt and p-Akt protein expression (*p* < 0.01 or *p* < 0.05) (Figure 6B,C), suggesting that osteogenic induction activates the phosphorylation process of the Akt protein. Following treatment with GBE18 cell-free supernatant (CFS), Akt protein expression was further increased by 1.44-fold relative to the ODM group (*p* < 0.05), and p-Akt expression was elevated by 1.75-fold (*p* < 0.01). This concurrent upregulation of Akt and p-Akt indicates that GBE18 CFS enhances the activation of the PI3K/Akt signaling pathway by promoting both Akt protein expression and phosphorylation levels. A quantitative analysis of active β-catenin bands (Figure 6D) demonstrated that the level of active β-catenin in the GBE18 group was 1.48 times higher than that observed in the ODM group (*p* < 0.05), indicating that GBE18 treatment effectively enhances β-catenin stability. As illustrated in the quantitative plot (Figure 6E), the CK group exhibited the lowest protein expression of Runx2. In contrast, the ODM group showed an increase in Runx2 expression compared to the CK group, and the GBE18 group exhibited a further significant upregulation of Runx2 expression by 1.55-fold (*p* < 0.05). These results suggest that osteogenic induction effectively promotes osteoblast differentiation, leading to increased protein expression of Akt, p-Akt, β-catenin, and Runx2. Moreover, GBE18 CFS further enhanced the expression of Akt, Active β-catenin, and Runx2, thereby amplifying signal transduction. These findings are consistent with the preceding results.

## 4. Discussion

Osteoporosis remains a significant global health challenge, particularly among postmenopausal women, as estrogen deficiency disrupts bone remodeling homeostasis by impairing osteoblast function and accelerating osteoclast-mediated bone resorption [18,19]. Current pharmacological interventions, such as bisphosphonates, primarily target osteoclast activity to mitigate bone loss; however, long-term administration is frequently associated with adverse effects [4,5]. Consequently, the development of safer therapeutic strategies that directly stimulate osteoblast-mediated bone formation has emerged as a critical research priority [20].

Accumulating evidence underscores the pivotal role of probiotics and their metabolites in modulating bone metabolism via the gut-bone axis [21]. Specific strains, such as *Lacticaseibacillus rhamnosus* GG and *Bifidobacterium longum*, have been shown to improve bone mineral density and microarchitecture in preclinical models [11,13]. Nevertheless, the direct effects of probiotic-derived soluble factors on osteoblast differentiation and the underlying molecular mechanisms remain poorly understood. In a preliminary screening of 38 probiotic strains preserved in our laboratory, *L. fermentum* GBE18 was identified as a candidate strain with prominent pro-osteogenic activity. In this study, we further demonstrated that the CFS of *L. fermentum* GBE18 promotes osteogenic differentiation and mineralization in MC3T3-E1 pre-osteoblasts, primarily through the coordinated activation of the Wnt/β-catenin and PI3K/Akt signaling pathways.

Biosafety is an essential requirement for any bioactive substance. Our CCK-8 assays demonstrated that GBE18 CFS, at concentrations ranging from 1% to 4% (*v*/*v*), exhibited no cytotoxic effects on MC3T3-E1 cells, consistent with the favorable biocompatibility observed in other probiotic fermentation products [22]. Importantly, while certain probiotic supernatants require specific dilution ratios to exert growth-promoting effects [14], GBE18 CFS increased the CCK-8 signal following extended exposure, in a concentration-independent manner. These findings suggest that GBE18 CFS may help maintain cellular activity and viability of MC3T3-E1 cells under the present experimental conditions. Alkaline phosphatase (ALP) is a recognized early indicator of osteogenic differentiation. Our study demonstrated that treatment with 2% GBE18 cell-free supernatant (CFS) significantly enhanced ALP activity at both 7 and 14 days following induction, aligning with the expected timeline of osteoblast differentiation. This finding parallels those reported for the extract of *Lacticaseibacillus paracasei* L30, which similarly increases ALP activity and subsequent mineralization [15]. In contrast, certain fermented substrates have been shown to inhibit ALP activity, highlighting the strain- and substrate-specific nature of probiotic-mediated osteogenic regulation [22]. Notably, the ability of GBE18 CFS to significantly promote ALP activity and mineralization in the absence of protein enrichment emphasizes its inherent osteogenic potential.

Osteogenic differentiation is governed by a complex network of signaling pathways. Our transcriptomic analysis demonstrated that treatment with GBE18 CFS significantly enriched DEGs within key osteogenesis-related pathways, most notably the Wnt and PI3K/Akt pathways. This dual-pathway activation represents a distinctive regulatory pattern observed with GBE18 CFS. While previous studies on probiotic metabolites have often concentrated on single pathways-for example, *Lacticaseibacillus casei* GKC1 primarily upregulates osteogenic genes such as *ALP* and *Runx2* without evident pathway synergy [23] our findings indicate a concurrent and significant enrichment of both the Wnt and PI3K-Akt cascades. Notably, key upstream regulators, including the Wnt pathway enhancer *Rspo2* and the PI3K-Akt kinase *Pdpk1*, were significantly upregulated, suggesting a mechanism driven by upstream activation and coordinated downstream effects.

The Wnt/β-catenin signaling pathway serves as a pivotal regulator of osteogenesis. Our transcriptomic data indicate that *Rspo2* and *Fzd* receptors are significantly upregulated in MC3T3-E1 cells treated with GBE18 CFS. According to established mechanisms, Rspo2 protects the Fzd5 receptor from ZNRF3/RNF43-mediated ubiquitination and degradation [24], thereby facilitating the accumulation of Fzd5 at the cell membrane. As a core receptor for Wnt3a, Fzd5 subsequently forms a complex with its co-receptor Lrp5/6 to activate Dvl proteins. This process recruits the destruction complex components—including Axin, GSK-3β, and APC—to the membrane, which prevents the degradation of β-catenin and promotes its cytoplasmic stabilization. Consequently, β-catenin translocates into the nucleus, where it binds to Tcf/Lef transcription factors and upregulates *Runx2* to promote osteogenesis.

Natural compounds, including γ-tocotrienol and arbutin, have been shown to facilitate osteoblast differentiation by stabilizing β-catenin and enhancing *Runx2* expression [25,26], consistent with our findings. The upregulation of *Fzd4* and *Fzd5* receptors observed in our RNA-seq data further corroborates the enhanced engagement of Wnt ligands and receptors, a mechanism similarly implicated in L-Quebrachitol-induced osteogenesis [27]. Simultaneously, the PI3K/Akt signaling pathway is integral to osteoblast survival, proliferation, and differentiation. Our Western blot analyses demonstrated that GBE18 CFS increased both total Akt and phosphorylated Akt (p-Akt) levels, indicating activation of this pathway. This observation is in agreement with previous studies where *Lacticaseibacillus paracasei* L30 extract and PTX3 activated the PI3K/Akt axis to promote osteogenic markers [15,28]. The interaction between the PI3K/Akt and Wnt/β-catenin pathways, potentially mediated through GSK-3β inhibition, likely contributes to the stabilization of β-catenin and the upregulation of *Runx2* expression, as evidenced by our protein-level data.

A significant finding of this study is the marked upregulation of the lncRNA *Malat1* following treatment with GBE18 CFS, to our knowledge, this is the first report demonstrating that probiotic-derived metabolites target Malat1 to promote osteogenesis While previous research has established the role of *Malat1* in bone biology—for instance, Qin et al. used in vivo knockout models to demonstrate that *Malat1* directly binds β-catenin in osteoblasts to enhance its transcriptional activity and maintain bone homeostasis [29], while Zhang et al. confirmed that *Malat1* modulates β-catenin expression and subcellular localization—our data extend these findings [30]. We reveal that GBE18 CFS-induced *Malat1* upregulation synergizes with the Wnt/β-catenin and PI3K/Akt pathways, likely by reinforcing β-catenin stabilization, to promote *Runx2* expression and osteogenic differentiation. This Malat1-mediated dual-pathway crosstalk adds a new layer of complexity to probiotic-induced osteogenesis that has not been previously described.

Collectively, these comparisons suggest that the pro-osteogenic effects of probiotics may be a shared feature across multiple strains, although the magnitude of the effect and the underlying mechanisms appear to be strain-specific. Compared with previous reports mainly focusing on bone mass improvement in vivo or changes in individual osteogenic markers, our study further provides transcriptomic and protein-level evidence that GBE18 CFS promotes osteogenesis through coordinated activation of the Wnt/β-catenin and PI3K/Akt pathways.

Despite these insights, the study has limitations that necessitate further investigation. Firstly, the specific bioactive compounds within GBE18 CFS responsible for the observed effects have not yet been identified. Secondly, the current findings are based solely on in vitro models; therefore, the observed effects cannot be directly extrapolated to clinical applications. Future studies employing osteoporotic animal models, such as ovariectomized mice, are crucial to confirm the bone-protective efficacy of GBE18 CFS in vivo and to investigate its potential interactions within the gut-bone axis. In our future work, we plan to further isolate and characterize the bioactive components in GBE18 CFS and evaluate their osteoprotective effects in relevant in vivo models.

## 5. Conclusions

In conclusion, this study demonstrates, based on transcriptomic analysis, quantitative real-time PCR, and Western blotting, that the CFS of *L. fermentum* GBE18 promotes osteogenic differentiation in MC3T3-E1 cells. The underlying molecular mechanism involves the coordinated activation of the Wnt/β-catenin and PI3K/Akt signaling pathways. Specifically, GBE18 CFS upregulates key regulators, including the Wnt enhancer Rspo2 and the PI3K/Akt kinase Pdpk1, thereby stabilizing β-catenin, enhancing Akt phosphorylation, and elevating the expression of the master osteogenic transcription factor *Runx2*. These findings provide mechanistic insights into the pro-osteogenic effects of GBE18 CFS and support the further exploration of probiotic-derived metabolites as candidate functional food ingredients for bone health support.

## Figures and Tables

**Table 1:** Effects of GBE18 CFS on DEGs in Wnt and PI3K Signaling Pathways.

Pathway	DEG(s)	Log2FC	FoldChange	*p*-Value
Wnt	*Wnt2b*	1.9474	3.8569	0.0431
	*Prickle2*	0.9303	1.9056	1.88 × 10^−10^
	*Tle2*	0.7237	1.6515	3.81 × 10^−8^
	*Ror1*	0.7198	1.6469	3.27 × 10^−6^
	*Plcb4*	0.5906	1.5059	2.42 × 10^−5^
	*Fzd5*	0.9149	1.8854	7.99 × 10^−10^
	*Fzd4*	0.6706	1.5918	0.0237
	*Rspo2*	1.0009	2.0013	2.75 × 10^−11^
	*Ep300*	0.6272	1.5445	2.63 × 10^−7^
	*Prkcg*	0.9073	1.8756	3.64 × 10^−6^
PI3K	*Pik3r1*	0.6886	1.6118	1.28 × 10^−11^
	*Irs1*	1.0622	2.0881	9.02 × 10^−14^
	*Pdpk1*	0.6922	1.6157	4.45 × 10^−11^
	*Tlr2*	1.2638	2.4012	1.12 × 10^−12^
	*Creb5*	0.7614	1.6951	0.0148
	*Tsc1*	0.6697	1.5908	6.47 × 10^−8^
	*Lama5*	1.1424	2.2075	0.0011
	*Itgb8*	0.8029	1.7446	8.97 × 10^−6^
	*Tnn*	0.6452	1.5640	7.09 × 10^−7^
	*Chrm1*	3.7519	13.4723	1.65 × 10^−7^

## Data Availability

The original contributions presented in this study are included in the article. Further inquiries can be directed to the corresponding authors.
